# Clinical and pathological tools for identifying microsatellite instability in colorectal cancer

**DOI:** 10.3325/cmj.2012.53.328

**Published:** 2012-08

**Authors:** Zoran Krivokapić, Srdjan Marković, Jadranka Antić, Ivan Dimitrijević, Daniela Bojić, Petar Svorcan, Njegica Jojić, Svetozar Damjanović

**Affiliations:** 1 Clinic for Digestive Surgery, Clinical Center of Serbia, Belgrade, Serbia; 2 Department for Gastroenterology and Hepatology, Zvezdara University Clinical Center, Belgrade, Serbia; 3 Institute of Endocrinology, Genetic laboratory, Clinical Center of Serbia, Belgrade, Serbia; 4 Medical Faculty, University of Belgrade, Belgrade, Serbia

## Abstract

**Aim:**

To assess practical accuracy of revised Bethesda criteria (BGrev), pathological predictive model (MsPath), and histopathological parameters for detection of high-frequency of microsatellite instability (MSI-H) phenotype in patients with colorectal carcinoma (CRC).

**Method:**

Tumors from 150 patients with CRC were analyzed for MSI using a fluorescence-based pentaplex polymerase chain reaction technique. For all patients, we evaluated age, sex, family history of cancer, localization, tumor differentiation, mucin production, lymphocytic infiltration (TIL), and Union for International Cancer Control stage. Patients were classified according to the BGrev, and the groups were compared. The utility of the BGrev, MsPath, and clinical and histopathological parameters for predicting microsatellite tumor status were assessed by univariate logistic regression analysis and by calculating the sensitivity, specificity, and positive (PPV) and negative (NPV) predictive values.

**Results:**

Fifteen out of 45 patients who met and 4 of 105 patients who did not meet the BGrev criteria had MSI-H CRC. Sensitivity, specificity, PPV, and NPV for BGrev were 78.9%, 77%, 30%, and 70%, respectively. MSI histology (the third BGrev criterion without age limit) was as sensitive as BGrev, but more specific. MsPath model was more sensitive than BGrev (86%), with similar specificity. Any BGrev criterion fulfillment, mucinous differentiation, and right-sided CRC were singled out as independent factors to identify MSI-H colorectal cancer.

**Conclusion:**

The BGrev, MsPath model, and MSI histology are useful tools for selecting patients for MSI testing.

Microsatellite instability (MSI) is a hallmark of mismatch repair (MMR) deficiency in hereditary nonpolyposis colorectal cancer (HNPCC). Because these carcinomas were observed to develop in the absence of polyposis, the term HNPCC was used instead of Lynch syndrome (LS). Moreover, HNPPC corresponds to at least two different entities, LS and type X familial colorectal cancer (1). LS is always characterized by germline defect of mismatch repair system and is associated with an increased lifetime risk for cancer, predominantly colorectal and endometrial cancer.

Testing for MSI is an important tool for identification of patients with hereditary colorectal cancer, because approximately 90% of HNPCC-associated colorectal tumors are characterized by MSI (2). Clinical criteria facilitated the identification of the molecular basis of HNPCC (3). Because the original criteria (Amsterdam criteria) were considered to be too restrictive (4,5), extended criteria were established (Amsterdam II criteria), which took into account other types of HNPCC-associated cancer, such as cancer of the endometria, small bowel, ureter, and renal pelvis (6-8). The use of the Amsterdam criteria achieved the original purpose of classifying HNPCC families but their limited sensitivity hampered decisions about which patients should undergo genetic testing. In 1996, an international workshop on HNPCC hosted by the National Cancer Institute outlined a set of recommendations, known as the Bethesda guidelines, for the identification of individuals with HNPCC who should be tested for MSI and/or genetic testing (4). The Bethesda guidelines initially proved to be highly sensitive, but were considered too indefinite and unsuitable for primary sequence analysis of MMR genes (4). The second HNPCC workshop revised these criteria and proposed a new set of recommendations, the revised Bethesda guidelines. The revised Bethesda guidelines (BGrev; Table 1) were published in 2004 (3). The purposes of revising the BGrev were better identification of patients at risk for hereditary colorectal cancer and identification if CRC is likely to be MSI-H. Four of the 5 criteria that make up these guidelines are based on family and personal history of Lynch-related tumors, whereas the fifth criterion is based on tumor histology (Table 1) (3).

**Table 1:** Revised Bethesda Guidelines (BGrev); just one of these criteria needed to be met (3)

**B1** Individuals diagnosed with colorectal cancer before the age of 50 y
**B2** Synchronous or metachronous colorectal or other HNPCC*-related tumors (which include stomach, bladder, ureter, renal pelvis, biliary tract, brain (glioblastoma), sebaceous gland adenomas, keratoacanthomas and carcinoma of the small bowel), regardless of age
**B3** Colorectal cancer with a high-microsatellite instability morphology^†^ that was diagnosed before the age of 60 y
**B4** Colorectal cancer with one or more first-degree relatives with colorectal cancer or other HNPCC-related tumors. One of the cancers must have been diagnosed before the age of 50 y (this includes adenoma, which must have been diagnosed before the age of 40 y)
**B5** Colorectal cancer with two or more relatives with colorectal cancer or other HNPCC-related tumors, regardless of age

*HNPCC – hereditary nonpolyposis colorectal cancer.

†Presence of tumor infiltrating lymphocytes, Crohn's-like lymphocytic reaction, mucinous/signet ring differentiation, or medullary growth pattern.

In 2007, Jenkins et al (9) published the MsPath model that used easily assessable clinicopathologic characteristics to capture all colorectal cancers with high frequency of microsatellite instability (MSI-H) presenting in patients younger than 60 years, the age group most likely to be associated with LS, while ruling out colorectal cancers that are highly unlikely to be MSI-H. This model (9) considers six clinicopathological features (with the corresponding coefficient): age at diagnosis (<50 years -0.7), anatomical site (cecum, ascending or transverse colon -1.6), histologic type (mucinous, signet ring, or undifferentiated -1.1), grade (poorly differentiated -0.6), Crohn-like reaction (present -0.5), and tumor-infiltrating lymphocytes (TILs) (present -2.1). Authors (9) have recommended a cutoff MsPath score of 1.0 to maximize the specificity while maintaining a high sensitivity, because it is important not to miss MSI-H cases.

MSI-H histology has been incorporated into the BGrev (third criterion BGrev, without age limit) for identifying patients with colorectal cancer for further genetic analysis.

The aim of the study was to evaluate clinical and pathological parameters in a regional cohort of Serbian unselected colorectal cancer patients who were tested for MSI in tumor tissue. We analyzed practical validity of revised Bethesda criteria, MSI histology, and MsPath model (9) for detection of MSI-H phenotype in CRC, by determination of sensitivity, specificity, positive (PPV), and negative (NPV) predictive value.

## Method

One hundred and fifty primary colorectal carcinomas were randomly selected for MSI testing and excised surgically at the Clinic for Digestive Surgery, Clinical Centre of Serbia, Belgrade, from January 2007-September 2010. The study was approved by the ethics committee of the Clinical Centre. Patients treated by preoperative radiotherapy or chemotherapy, those with inflammatory bowel disease, or a known history of familial adenomatous polyposis were excluded. Family history data were obtained through an interview with each patient at hospital admission. Patients who fulfilled the Amsterdam criteria were also excluded. Fresh representative tissue samples from all 150 tumors were immediately frozen at -80°C and tested for MSI. The genomic DNA was extracted using QIAamp DNA Mini Kit (Qiagen, Hilden, Germany) according to the manufacturer’s protocol (10).

Five mononucleotide markers, BAT-25, BAT-26, NR-21, NR-22, and NR-24, were coamplified in a single pentaplex polymerase chain reaction (PCR) mix containing QIAGEN Multiplex PCR Kit, five fluorescent primers set in a final concentration of 0.25 μmol/L for each primer, and 100 ng of DNA, in the previously described conditions (11). The size of PCR products and the corresponding fluorescent label Gene Scan 500LIZ Size Standard were analyzed in ABI PRISM 3130 Genetic Analyzer (Applied Biosystems, Foster City, CA, USA) using Gene Mapper Software, version 3.7. The size of PCR products and the corresponding fluorescent labels were chosen so as to allow simultaneous analysis of normal-sized alleles, with the smaller-sized alleles containing deletions typically seen in MSI-H tumors. Tumors were classified as MSI-H if three or more out of five markers showed MSI and as microsatellite stable/with low frequency of microsatellite instability (MSS/MSI-L) if none or fewer than three markers showed MSI. Patients were divided into two groups based on revised Bethesda criteria (BGrev + patients who fulfilled any of the criteria and BGrev- patients who did not fulfill any of the criteria). The following pathological parameters were examined independently by an experienced pathologist: mucin production graded as present or none, tumor-infiltrating lymphocytes (TILs) graded also as present (at least 5 per high power field) or none, and tumor differentiation graded as poor, moderate, and good. Sensitivity, specificity, PPV, NPV of BGrev, MSI histology, MsPath model, and pathological parameters (presence of any mucin, TILs, poor differentiation) for detecting MSI-H CRC were calculated.

### Statistical analysis

Results are expressed as mean ± standard deviation for parametric data and counts for non-parametric data. Statistical analysis was performed between groups using independent samples *t*-test to analyze numerical parameters (of normally distributed variables), while asymptotic χ^2^ and χ^2^ likelihood ratio tests were used for non-parametric data. Univariate logistic regression analysis was performed to identify significant predictors of MSI status and to calculate the odds ratio (OR). The utility of different parameters at predicting MSI status was compared by assessing the sensitivity, specificity, positive (PPV) and negative (NPV) predictive values, which were calculated using standard definition (12). Statistical analysis was performed with SPSS 15.0 software (SPSS Inc., Chicago, IL, USA). All *P* values lower than 0.05 were considered as significant.

## Results

A total of 150 patients were enrolled (60 women, 90 men; mean age at diagnosis, 61 ± 10.3 years). Forty seven patients had left-sided colorectal cancer (in the descending and sigmoid colon), 28 had right-sided colorectal cancer (in the transverse colon, ascending colon, or cecum), and 75 had rectal cancer. The cohort included synchronous or metachronous colorectal cancer in 5 patients.

Forty five of the 150 patients with colorectal cancer (30%) fulfilled at least one of the five revised Bethesda criteria (Table 1), whereas 105 patients (70%) fulfilled none. The most commonly fulfilled criteria were B1 and B3 (Table 2). The distribution of sex, Union for International Cancer Control (UICC) tumor stage, and pathological grading were similar in the patients who did and did not fulfill the revised Bethesda criteria (Table 3).

**Table 2:** Number of patients who met the revised Bethesda criteria and microsatellite instability status of these patients

	B1(<50 y)	B2	B3	B4	B5	B1+B3	B3+B4	B1+B3+B5	Ʃ
**Number of patients**	**16**	**5**	**11**	**4**	**2**	**4**	**2**	**1**	**45**
High frequency of microsatellite instability	**0**	**1**	**4**	**4**	**2**	**1**	**2**	**1**	**15**
Microsatellite stable **/**low frequency of microsatellite instability	**16**	**4**	**7**	**0**	**0**	**3**	**0**	**0**	**30**

**Table 3:** Patients’ clinical characteristics, according to fulfillment (BGRev+) or nonfulfillment (BGRev-) of at least one criterion of the revised Bethesda guidelines

Variable	BGRev- (n = 105)	BGRev+ (n = 45)	All patients (n = 150)	*P*
**Women/men**	43/62	17/28	60/90	0.351*
**Mean age ± standard deviation (range)**	65.1 ± 10.3	51.6 ± 9.9	61.08 ± 10.3	<0.001^†^
**Localization:**				0.712*
rectum	52	23	75	
left colon	34	13	47	
right colon	19	9	28	
**Patients with synchronous or metachronous colorectal cancer**	0	5	5	
Union for International Cancer Control disease stage:				0.572*
I	7	6	13	
II	38	14	52	
III	46	19	65	
IV	14	6	20	

*χ^2^ test.

^†^*t*-test.

**Figure 1:**
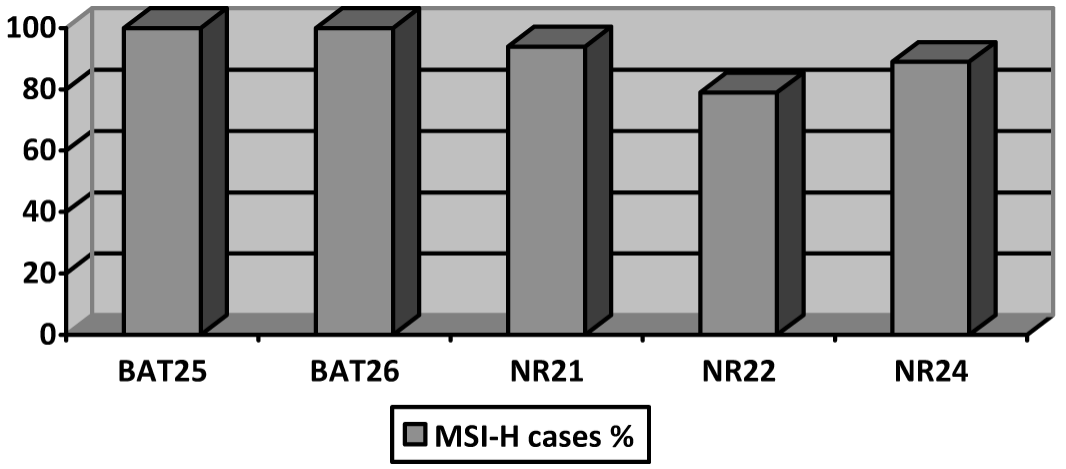
Performance of microsatellite markers to detect microsatellite instability (MSI, N = 19). The microsatellite markers showed MSI in the following percentages of high-level MSI (MSI-H) cases: BAT25, 100%; BAT26, 100%; NR21, 94%; NR22, 79%; and NR24 89%.

Microsatellite instability was detected in 19 of the 150 patients with colorectal cancer (12.6%). The results obtained with the individual microsatellite markers are summarized in Figure 1. The mononucleotide repeat markers BAT26 and BAT25 were most sensitive. Fifteen of the 45 patients who met the revised Bethesda criteria (30%) and 4 of the 105 patients who met none of the revised Bethesda criteria (3.4%) had a tumor with microsatellite instability. In the group of patients with synchronous or metachronous colorectal cancer, one patient had MSI-H, one had MSI-L (BAT25), and 3 had MSS CRC. Characteristics of patients with CRC and MSI are shown in Tables 3,4,5.

**Table 4:** Patients’ histopathological characteristics and microsatellite instability (MSI) status, according to fulfillment (BGRev+) or nonfulfillment (BGRev-) of at least one criterion of the revised Bethesda guidelines

Variable	BGRev- (n = 105)	BGRev+ (n = 45)	All patients	*P*	Odds ratio (95% confidence interval)
**Tumor infiltrating lymphocytes:**				0.130*	
1 (present)	38	18	56		
2 (absent)	67	27	94		
**Differentiation:**				0.632*	
1 (undifferentiated and poorly differentiated)	11	4	15		
2 (moderately differentiated)	45	20	65		
3 (well differentiated)	49	21	70		
**Mucin production:**				0.023*	3.1 (4.1-4.4)^†^
present	77	21	98		
absent	28	24	52		
**MSI status:**				0.0001*	7 (2.5-19.9)^†^
High frequency of microsatellite instability	4	15	19		
Microsatellite stable/low frequency of microsatellite instability	101	30	131		

*χ^2^ test.

^†^Mantel-Haenszel test.

**Table 5:** Characteristics of patients with colorectal cancer (CRC) and microsatellite instability (MSI)

Patient	Sex	Age at CRC diagnosis	Colorectal tumors	Location of CRC	Tumor node metastases stage, grade	Family history of HNPCC* related cancer	Revised Bethesda criteria met	Histology suggestible for MSI
1	m	44	1	rectum	T2N0M0,GI	no	B1, B3	yes
2	f	64	1	**ascending colon**	T2N0M0,GIII	yes	B4	yes
3	m	68	1	rectum	T3N1M0,GII	yes	B4	yes
4	m	72	2	**cecum** and rectum	T2N0M0,GII T3N0M0,GIII	no	B2	no
5	m	52	1	**cecum**	T4bN2M0,GII	yes	B3	yes
6	m	56	1	rectum	T3N0M0, GII	yes	B3,B4	yes
7	m	64	1	**cecum**	T2N0M0,GI	yes	B4	yes
8	f	63	1	**cecum**	T2N1M0, GII	yes	B5	yes
9	m	41	1	rectum	T1N0M0GIII	yes	B1,B3,B5	no
10	m	58	1	**ascending colon**	T3N0M0, GII	no	B3	no
11	m	66	1	descending colon	T1N0M0, GII	yes	B5	no
12	m	57	1	rectum	T3N1M0, GII	no	B3	yes
13	m	57	1	**ascending colon**	T3N1M0, GIII	no	B3	yes
14	m	59	1	sigmoid colon	T4aN0M0, GII	no	B3	yes
15	m	59	1	descending colon	T2N0M0,GI	yes	B4,B3	yes
16	f	70	1	**transverse colon**	T3N0M0, GII	no	-	no
17	f	76	1	**ascending colon**	T4bN1M1,GII	no	-	no
18	m	71	1	rectum	T3N1M0, GI	no	-	no
19	m	68	1	**ascending colon**	T3N0M0,GII	no	-	no

*HNPCC – hereditary nonpolyposis colorectal cancer.

The sensitivity of the revised Bethesda criteria to detect microsatellite instability in our cohort was 79% (confidence interval [CI], 54% to 93%), and the specificity was 77% (CI, 68.7% to 83.7%). Positive and negative predictive value was 30% and 70%, respectively. When the age limit of 45 years was used in the first criterion of BGrev (instead 50 years), the specificity was 89%, while sensitivity, PPV, and NPV did not significantly change. We estimated the utility of the B3 criterion, MSI histology (B3 criterion without age limit, defined by presence of TILs, Crohn's-like lymphocytic reaction, mucinous/signet ring differentiation, or medullar growth pattern), TILs, mucinous and poor tumor differentiation, family history of cancers (B4, B5 criteria together) for identification MSI-H tumors. Specificity, sensitivity, PPV, and NPV for these parameters are given in the Table 6. MsPath model with recommended cut-off score of 1.0 identified 84% of our MSI-H tumors (patients below the age of 60 years). The sensitivity and specificity were 86% and 85%, respectively.

**Table 6:** Sensitivity, specificity, positive and negative predictive value of histological and clinical features in predicting microsatellite instability (MSI)

Variable	Sensitivity	Specificity	Positive predictive value	Negative predictive value
**B3 criterion**	0.44	0.91	0.13	0.87
**MSI histology**	0.79	0.85	0.20	0.80
MsPath score*	0.86	0.85	0.18	0.82
**Tumor infiltrating lymphocytes**	0.52	0.65	0.37	0.63
**Any mucinous differentiation**	0.63	0.69	0.34	0.65
**Poor differentiation**	0.27	0.89	0.13	0.88
**Family history of cancers (B4.B5)**	0.64	0.92	0.13	0.87
**Revised Bethesda guidelines**	0.78	0.77	0.3	0.7
**Revised Bethesda guidelines (modified B1, age limit <45 y)**	0.78	0.89	0.19	0.80

*Cut-off value = 1.

Eight clinicopathological features were included into univariate logistic regression analysis – diagnostic age lower than 60 years, male sex, right-sided colon cancer, poor differentiation, mucinous differentiation, the presence of TILs, lower disease stage (I and II UICC stage), and any BGrev criterion fulfillment. Any BGrev criterion fulfillment, mucinous differentiation, and right-sided CRC were singled out as independent factors to identify MSI-H colorectal cancer (Table 7).

**Table 7:** Univariate logistic regression analysis of clinical and histopathological predictors of microsatellite instability

Variable	Odds ratio	95% confidence interval	*P*
Male sex	2.59	0.8-8.2	0.125
Age less than 60 years	1.74	0.4-3.6	0.344
**Right-sided CRC**	**8.5**	**2.7-26.7**	**0.001**
Poor and well differentiation	2.18	0.8-7.3	0.181
**Any mucinous differentiation**	**3.272**	**1.2-8.7**	**0.040**
TIL>5/HPF	2.21	0.8-5.4	0.158
Lower disease stage (UICC stage; I and II)	1.06	0.3-2	0.918
Any BGrev criterion fulfillment	6.99	2.5-19.9	0.001

*Abbreviations: CRC – colorectal cancer**;** TIL **–** tumor infiltrating lymphocytes; HPF – high-powered field; UICC – Union for International Cancer Control staging; BGrev – Revised Bethesda guidelines.

Pathological parameters (tumor differentiation, mucin production, and TILs) were compared between BGrev + and BGrev- patients. There were no significant differences between the groups, when differentiation and TILs were used as grouping criteria. Mucinous carcinomas were significantly more frequently present in BGrev + group (odds ratio 3.14; 95% CI, 1-4.4; *P* = 0.02) (Table 4).

## Discussion

In this prospective study of unselected and consecutively diagnosed CRC patients, we assessed the performance of currently used clinical guidelines (BGrev) against a molecular tumor marker (MSI). The frequency of high microsatellite instability phenotype in our cohort was 12.6%. Nearly 80% of MSI-H CRCs were identified using BGrev, and the specificity of MsPath model and MSI histology was higher than that of BGrev. Among other clinical and pathological features, mucinous differentiation and right-sided CRC were independent factors for identifying MSI-H colorectal cancer.

In the early 1990s, the genetic defect responsible for LS was identified as a germline mutation in one of the DNA MMR genes with the consequence of a microsatellite instability phenotype (13). Introducing the MSI determination as an initial screening test for CRC enables the molecular detection of LS in large populations. MSI-H has been shown to have a dominant impact on the global molecular phenotype in CRC. MSI-H CRC shows distinct clinicopathological features, including both better prognosis (14,15) and reduced response to 5-fluorouracil/leucovorin (5-FU) adjuvant chemotherapy (16,17). Moreover, patients with MSS tumors (especially stage III cancers) seem to benefit most from adjuvant 5FU chemotherapy. Conversely, patients with MSI tumors and more specifically those with stage II cancer, do not seem to benefit from adjuvant chemotherapy (16,17). Both the MSI test and immunostaining have been shown to be highly effective for selecting patients who should be tested for hMSH2/hMLH1 germline mutations (18). In this study, we did not include patients who fulfilled the Amsterdam criteria for HNPCC, because in our and other opinions (2,6), patients with CRCs belonging to HNPCC families should be proceeded immediately to MMR gene mutation analysis. These patients do not need the MSI analysis.

In a recent study (19), the usefulness of BGrev for MSI prediction was assessed with and without B3. In this cohort, 2/3 patients with MSI-H were identified by the BGrev, resulting in a sensitivity and specificity of 66.7% and 50.9%, respectively. The sensitivity of MSI histology in the same study (19) was 82.5%, while the specificity was lower, 27.1%. In our cohort, MSI histology had lower sensitivity (78.9%) and higher specificity (84.7%) than BGrev. In 2007, Jenkins et al (9) published the MsPath model for predicting MSI in CRC. This model did not include family history, but included histology features described in the B3 criterion (Table 1) with tumor localization and age at diagnosis. MsPath model is only applied to patients diagnosed before the age of 60 years. MsPath score ≥1.0 had a sensitivity of 93% and a specificity of 55% for MSI-H tumors (9). Applied MsPath model with recommended cut-off score of 1.0 in our cases had an increased sensitivity of 86%. The specificity of MsPath model and BGrev in our cohort was similar. Differences, especially in specificity, between our and other studies can be explained by the fact that our cohort was unselected. A large number of patients with MSI-H CRCs are older than 60 years and they would be missed by this model. In our study, more than half patients with MSI-H tumor were older than 60 years. It is likely that these cases are sporadic MSI-H CRCs. In sporadic MSI-H CRC, MSI occurs due to MLH1 genes promoter methylation. Five patients with MSI-H CRC were between 50 and 60 years and they all met B3. The possibility of LS diagnosis in these cases is higher. Moreover, the analysis of the MMR defect discriminating sporadic from hereditary MSI-H cases needs to be developed in order to be able to recognize Lynch cases in Serbia.

An important finding was that the specificity of revised Bethesda criteria could be increased using the lower age limit in the B1 criterion (<45 years instead <50 years) as used in the previous Bethesda criteria. Twenty one patients in our cohort were younger than 50 years. Among these patients, only two (10%) had MSI-H tumors and both were younger than 45 years. When the age limit in the B1 criterion was 45 years, BGrev were more specific. However, this observation is only hypothetical and is not applicable in practice as long as germline mutation is not determined to confirm the final diagnosis of LS.

Regarding the patients presenting with synchronous or metachronous colonic tumors, four patients were MSS and only one was MSI-H, and this is another reason for the low PPV and higher specificity of the BGrev in our study population (great number of false positives).

Another study (20) concluded that tumor localization, rather than MSI histology, might have a key role in detecting CRC with MSI-H phenotype. These authors suggested that in a future revision of criteria for MSI testing, tumor localization should play as great a role as age at the onset of the CRCs, and the Bethesda criteria should be broadened to include patients 51-60 years old with proximal colon cancer. Our findings support this claim.

We found that identification of mucinous histology, even if seen only focally, was significant, independent predictor of MSI-H CRC. Others (19,21) have reported similar findings. In one study (22), the specificity of TILs for MSI-H CRC reached 98.2%, while the sensitivity was low (33.3%). Our results showed lower TILs specificity and higher sensitivity. In our study, TILs was not an independent predictive factor for MSI-H CRC. Results from other studies (19,23,24) were different. Tumor differentiation was another feature that was not an independent predictor of MSI-H in our study but was in others (25-27). Greenson et al (21) considered well and poor differentiated tumors together, which indicated an increased likelihood of MSI. We assessed the grade using both of these models, and no significance was found. Poor differentiation proved to be a specific parameter for MSI-H CRC. Proximal tumors were more likely to show MSI-H phenotype than distal tumors, which concurs with the results from other studies (28,29).

The main limitation of our study was the inability to analyze germline mutations of MMR genes for MSI-H tumors. Another limitation was the fact that only routine histopathological parameters that were included in the BGrev and MsPath model (9) were analyzed, so we were not able to examine the validity of newer proposed models for the prediction of MSI-H (19,21). Also, the selection of patients based on their age was not made, so the validity of clinical and histopathological parameters can be reliably observed.

In conclusion, BGrev are useful to select patients at risk for hereditary cancer, by increasing the detection rate of microsatellite instability as MSI histology. MsPath model proved to be more sensitive than BGrev, with similar specificity. Moreover, BGrev, MsPath model, and MSI histology are useful tools for selecting patients with CRC for MSI testing.
